# MRI-Based Radiomics and Urine Creatinine for the Differentiation of Renal Angiomyolipoma With Minimal Fat From Renal Cell Carcinoma: A Preliminary Study

**DOI:** 10.3389/fonc.2022.876664

**Published:** 2022-05-26

**Authors:** Lian Jian, Yan Liu, Yu Xie, Shusuan Jiang, Mingji Ye, Huashan Lin

**Affiliations:** ^1^ Department of Radiology, Hunan Cancer Hospital, The Affiliated Cancer Hospital of Xiangya School of Medicine, Central South University, Changsha, China; ^2^ Department of Urological Surgery, Hunan Cancer Hospital, The Affiliated Cancer Hospital of Xiangya School of Medicine, Central South University, Changsha, China; ^3^ Department of Pharmaceuticals Diagnosis, General Electric (GE) Healthcare, Changsha, China

**Keywords:** minimal fat angiomyolipoma, renal cell carcinoma, radiomics, nomogram, diagnosis

## Abstract

**Objectives:**

Standard magnetic resonance imaging (MRI) techniques are different to distinguish minimal fat angiomyolipoma (mf-AML) with minimal fat from renal cell carcinoma (RCC). Here we aimed to evaluate the diagnostic performance of MRI-based radiomics in the differentiation of fat-poor AMLs from other renal neoplasms.

**Methods:**

A total of 69 patients with solid renal tumors without macroscopic fat and with a pathologic diagnosis of RCC (n=50) or mf-AML (n=19) who underwent conventional MRI and intravoxel incoherent motion diffusion-weighted imaging (IVIM-DWI) were included. Clinical data including age, sex, tumor location, urine creatinine, and urea nitrogen were collected from medical records. The apparent diffusion coefficient (ADC), pure diffusion coefficient (D), pseudodiffusion coefficient (D*), and perfusion fraction (*f*) were measured from renal tumors. We used the ITK-SNAP software to manually delineate the regions of interest on T2-weighted imaging (T2WI) and IVIM-DWI from the largest cross-sectional area of the tumor. We extracted 396 radiomics features by the Analysis Kit software for each MR sequence. The hand-crafted features were selected by using the Pearson correlation analysis and least absolute shrinkage and selection operator (LASSO). Diagnostic models were built by logistic regression analysis. Receiver operating characteristic curve analysis was performed using five-fold cross-validation and the mean area under the curve (AUC) values were calculated and compared between the models to obtain the optimal model for the differentiation of mf-AML and RCC. Decision curve analysis (DCA) was used to evaluate the clinical utility of the models.

**Results:**

Clinical model based on urine creatinine achieved an AUC of 0.802 (95%CI: 0.761-0.843). IVIM-based model based on *f* value achieved an AUC of 0.692 (95%CI: 0.627-0.757). T2WI-radiomics model achieved an AUC of 0.883 (95%CI: 0.852-0.914). IVIM-radiomics model achieved an AUC of 0.874 (95%CI: 0.841-0.907). Combined radiomics model achieved an AUC of 0.919 (95%CI: 0.894-0.944). Clinical-radiomics model yielded the best performance, with an AUC of 0.931 (95%CI: 0.907-0.955). The calibration curve and DCA confirmed that the clinical-radiomics model had a good consistency and clinical usefulness.

**Conclusion:**

The clinical-radiomics model may be served as a noninvasive diagnostic tool to differentiate mf-AML with RCC, which might facilitate the clinical decision-making process.

## Introduction

Angiomyolipoma (AML) is the most common benign solid renal tumor with an estimated prevalence of 0.1% of men and 0.22% of women without tuberous sclerosis (1). In most cases, classic AMLs can be visually interpreted by identifying the intratumoral macroscopic fat component on computed tomography (CT) or magnetic resonance imaging (MRI) scans. However, around 4.5% of this type of tumors are classified as minimal fat AML (mf-AML) due to no microscopically detectable fat or the intratumoral fat is too small to be observed by radiologists; as a result, misinterpretation of mf-AMLs as renal cell carcinomas (RCCs) results in unnecessary surgery in some patients (2–6). Thus, preoperative differentiation between mf-AML and RCC is of great importance for clinical decision-making, however, accurate, reliable, and non-invasive assessment tools are lacking.

Research on the differential diagnosis of mf-AML is difficult to accumulate cases, because its frequency is significantly lower than that of other common renal tumors. Prior studies have shown several imaging findings (mainly from CT) may be helpful for diagnosis of mf-AML, such as an oval shape without capsule (7), homogeneous hypointensity on T2-weighted MR images (8), higher attenuation than renal parenchyma (9), tumor-to-cortex enhancement ratio (10), and prolonged enhancement pattern (9). The diagnostic accuracy of standard MRI with opposed-phase and in-phase gradient-echo (GRE) sequences for the differentiation of mf-AML and RCC was poor (11). Accordingly, some functional MRI techniques have been investigated, including diffusion-weighted imaging (DWI) (11–16), chemical-shift MRI (17, 18), diffusion kurtosis imaging (19). However, differentiation of mf-AML from RCC remains challenging due to limitations of low inter-observer agreement and unsatisfactory diagnostic accuracy and specificity.

With advances in computational hardware and mathematical algorithms, there is an increasing trend in acquiring quantitative information from daily medical images and correlating it with outcomes (20). Although deep learning is currently the mainstream of diagnostic imaging method, it should be noted that the other techniques such as radiomics, can be useful for diseases that are difficult to accumulate cases. Radiomics refers to an emerging technology of high-throughput extraction of a large number of quantitative descriptors from standard-of-care medical images, and it then translates image data into high-dimensional and mineable data *via* a variety of computer-aided algorithms, such as machine learning (21). A growing body of evidence has shown the potential of quantitative radiomics features in uncovering tumor characteristics that fail to be appreciated by the naked eye (22). To date, radiomics has been widely applied in precision diagnosis, treatment response evaluation, and prognosis prediction across many types of cancer (23–25).

The purpose of our study, therefore, was to prospectively evaluate the diagnostic performance of MRI-based radiomics in the differentiation of AML with minimal fat from other renal neoplasms by using pathologic reports as the reference standard.

## Materials and Methods

### Patients

This retrospective study was approved by our institutional review board; written informed consent was waived. An initial search of our institutional pathology database yielded the records of patients who underwent surgery for a renal mass between January 2013 and May 2021. After that, we identified the patients who met the following inclusion criteria: (1) pathologically confirmed RCC or AML; (2) single tumor ≥1 cm; (3) available MRI data, including conventional MRI and intravoxel incoherent motion (IVIM) DWI data before surgery; (4) no macroscopic fat component within the renal tumor identified on conventional MRI images, which were independently reviewed by two radiologists with more than 10 years of experience in genitourinary radiology; and (5) patients were not treated with chemotherapy or radiotherapy prior to surgery. 807 patients were excluded for the following reasons: (1) the maximum diameter of the tumor was <1.0 cm (n=18); (2) other types of renal tumors except for RCC and AML, such as sarcoma, cystic renal cell carcinoma (n=48); (3) AML with intratumoral macroscopic fat (n=30); (4) no preoperative MRI or IVIM-DWI images available (n=701); (5) inadequate image quality for analysis due to motion artifacts (n=10). Finally, a total of 69 patients with solid renal tumors without macroscopic fat and with a pathologic diagnosis of RCC (n=50) or mf-AML (n=19) were finally included. The RCCs consisted of seven papillary RCC, one chromophobe RCC, and 42 clear cell RCC (ccRCC). Clinical characteristics including age, sex, tumor location, urine creatinine, and urea nitrogen were collected from medical records.

### MRI Image Acquisitions

All MR images were acquired using a 1.5T MRI unit (Optima MR360, GE Healthcare, Waukesha, WI, USA). The conventional MRI protocols were listed as follows: (1) axial T1-weighted dual-echo in-phase and out-of-phase sequences: time of repetition/time of echo (TR/TE) = 205/4.2 ms; slice thickness = 4 mm; interslice gap = 1 mm; field of view (FOV) = 380 × 342 mm; matrix = 256 × 160; number of excitations (NEX) = 1. (2) axial T2-weighted fast spin-echo (FSE) images with fat suppression: TR/TE = 6000/86.4 ms; slice thickness = 4 mm; interslice gap = 1 mm; FOV = 380 × 342 mm; matrix = 320 × 192; NEX = 2. (3) axial and coronal contrast-enhanced T1-weighted fast spoiled gradient echo (FSPGR) with fat suppression: TR/TE = 165/2.3 ms; slice thickness = 4 mm; interslice gap = 1 mm; FOV = 400 × 400 mm; matrix = 384 × 192; NEX = 2. The contrast medium Gadodiamide (Omniscan^®^, GE Healthcare) was administered intravenously at a dose of 0.1 mmol/kg of body weight. IVIM-DWI scan was performed using a single-shot diffusion-weighted spin-echo echo-planar sequence. The DWI images were obtained prior to the injection of contrast agent with TR/TE of 6000/81.7 ms, slice thickness of 4 mm, interslice gap of 1 mm, FOV of 380 × 304 mm, matrix of 128 × 130, and NEX of 4. Parallel imaging was used with an acceleration factor of 2. Twelve b values (0, 20, 30, 50, 80, 100, 150, 200, 400, 600, 800, and 1000 s/mm^2^) were used in three orthogonal diffusion directions. A lookup table of gradient direction was modified to allow multiple b-value measurements in one series.

### IVIM-DWI Image Postprocessing

IVIM parameter values were calculated using the following equation:


 (1) $$S_{b}/S_{0}\;=\;f\;\exp\;\left(-bD*\right)\;+\;\left(1\;–f\right)\;\text{exp}\;\left(-bD\right)$$

Where S_b_ and S_0_ are the signal intensities without and at a given b value, respectively. D is the true water molecule difusion coefcient; D* is the perfusion coherence difusion coefcient, i.e., pseudodispersion, which can refect changes in blood perfusion. *f* is the perfusion-related volume fraction, representing the volume ratio of the difusion caused by the microcirculation perfusion efect in the overall difusion efect of the voxel.

**Figure 1:**
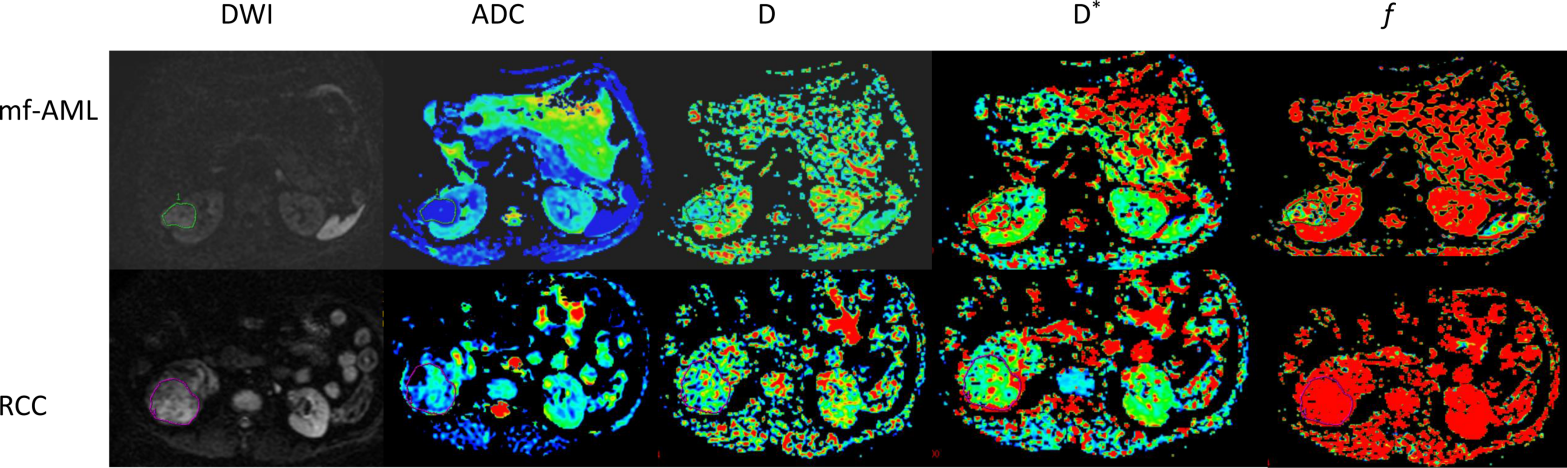
IVIM-DWI images of mf-AML and RCC. The tumors were displayed in DWI images. The D, D*, and *f* maps were obtained from IVIM of mf-AML in the top low and RCC in the bottow row, respectively. Outlines indicate the tumor region.

The IVIM sequence image raw data were transmitted to the functool software and MADC postprocessing software of GE’s ADW 4.5 workstation for image postprocessing and analysis, and the IVIM parametric images were obtained (**Figure 1**). The values of IVIM parameters were independently measured by two radiologists with 15 years and 20 years of diagnostic imaging experience. The detailed method for ROI delineation has been described in a previous study (26).

### Tumor Segmentation, Feature Extraction, and Feature Selection

**Figure 2:**
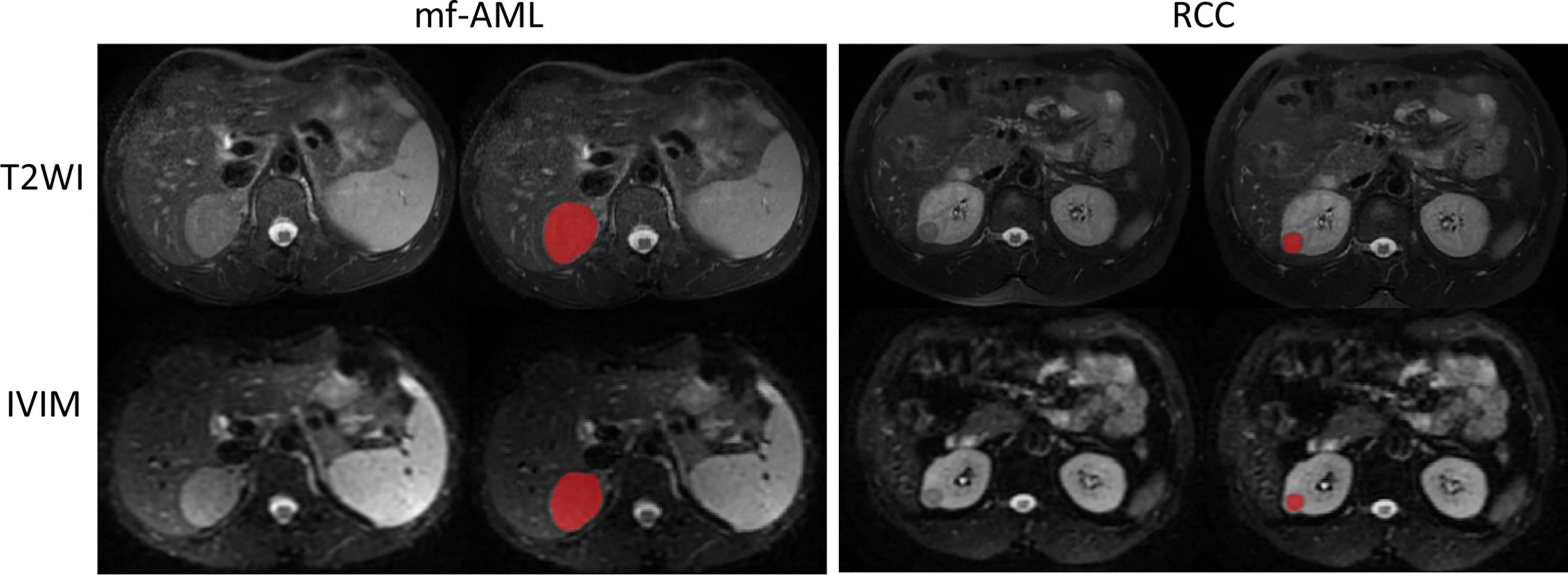
Example of tumor segmented by the radiologist. The mf-AML and RCC were segmented on the T2-weighted image but the same segmentation has been copied to IVIM-DWI (b = 1000 s/m^2^) image.

*Image segmentation*: We used an open-source ITK-SNAP software (version 3.6.0, www.itksnap.org) for manual segmentation on T2-weighted and IVIM-DWI (b = 1000 s/m^2^) MR images (**Figure 2**). Tumor segmentation was performed on the largest cross-sectional area of the tumor by a radiologist (with 15-year experience) and subsequently reviewed by a board-certified radiologist (20-year experience).

*Feature extraction*: The radiomics feature extraction was performed by AK software (Artificial Intelligence Kit, GE life Sciences, AA R&D team, Shanghai, China) equipped with pyradiomics, which was consistent with the standards set by the Image Biomarker Standardization Initiative. In total, 396 radiomics features were extracted from each MRI sequence, including four groups: 1) Histogram (n=42); 2) Gray-level co-occurrence matrix (GLCM) (n=108); 3) Gray-level run length matrix (GLRLM) (n=226); and 4) Shape (n=20). GLCM and GLRLM in four directions (0°, 45°, 90°, 135°) and three displacements (1, 4, 7) were calculated to describe patterns or the spatial distribution of voxel intensities. The description of the extracted radiomics features is shown in **Supplementary Material**.

*Feature* pre-*processing and feature selection*: Before feature selection, two steps of feature pre-processing were done: step 1—outliers were replaced by the median of the same feature; step 2—Z-score normalization was conducted in the training cohort to eliminate the difference in the value scale of extracted features (14). First, intraclass correlation coefcient (ICC) was used to quantify the stability of each radiomic feature. A feature was considered stable if ICC was higher than 0.75. Second, Pearson correction (PCC) analysis was then used to assess the correlation between radiomics feature pairs; a PCC of 0.99 was usually used to eliminate the redundancy. Finally, the least absolute shrinkage and selection operator (LASSO) algorithm was used for further radiomics feature selection.

### Development of Multiple Diagnostic Models

The significant predictors of clinical and IVIM parameters were identified by univariate and multivariate logistic regression analyses (P<0.05). The clinical model, IVIM-based model, T2WI-radiomics model, IVIM-radiomics model, combined radiomics model, and clinical-radiomics model were constructed by stepwise logistic regression. A nomogram was built based on the results of clinical-radiomics model. Considering the small sample size of the study, we didn’t split the entire data into training and validation sets but applied 5-fold cross-validation to avoid overfitting. The calibration curve and Hosmer-Lemeshow statistic were done to evaluate the agreement between the predicted probability and actual diagnosis. The decision curve analysis (DCA) was performed to determine the clinical usefulness of the nomogram by quantifying the net benefits at different threshold probabilities (27).

### Statistical Analysis

The statistical analyses were performed using R software (version 4.0.1; http://www.R-project.org) and Python software (version 3.7, http://www.python.org). As for continuous variables, data were expressed as mean ± standard deviation (SD) or median (interquartile range, IQR), while for categorical variables, data were expressed as counts and percentages (n, %). Continuous and categorical variables were compared by t-test, Mann-Whitney U test, Chi-square, if appropriate. The diagnostic performance of models was evaluated by the receiver operator characteristic curve (ROC) analysis. DeLong’s test was used to compare the AUCs of the two models. A two-tailed P<0.05 indicated statistical significance.

## Results

### Patient Characteristics

**Table 1** shows the characteristics of patients and tumors. There were no significant differences in age, sex, laterality, and urea nitrogen (all P values >0.05). The urine creatinine of RCC was significantly lower than that of mf-AML (87.5 ± 29.9 vs. 112.2 ± 12.4, P<0.001).

**Table 1:** Characteristics of patients and tumors.

	mf-AML (n=19)	RCC (n=50)	P-value
Age (years)	55.3 ± 11.8	51.4 ± 12.1	0.227
Sex			0.058
Male	15 (78.9)	27 (54.0)	
Female	4 (21.1)	23 (46.0)	
Laterality			0.558
Left kidney	8 (42.1)	25 (50.0)	
Right kidney	11 (57.9)	25 (50.0)	
Urine creatinine (μmol/L)	112.2 ± 12.4	87.5 ± 29.9	<0.001
Urea nitrogen (mmol/L)	6.6 ± 2.1	5.4 ± 2.1	0.032

### Comparison of the IVIM-DWI Parameters Between Two Groups

No significant differences were observed in ADC, D, and D* between two groups (P=0.296 0.439, and 0.185, respectively), only f value was significantly different between mf-AML and RCC (P=0.004) (**Table 2**).

**Table 2:** Comparison of the IVIM-DWI parameters between mf-AML and RCC groups.

Parameters	mf-AML (n=19)	RCC (n=50)	P-value
ADC (× 10^-3^ mm^2^/s)	1.99 ± 0.43	2.21 ± 0.47	0.296
D (× 10^-3^ mm^2^/s)	1.54 ± 0.31	1.63 ± 0.45	0.439
D* (× 10^-3^ mm^2^/s)	12.63 ± 2.93	15.41 ± 8.81	0.185
*f*	0.49 ± 0.21	0.36 ± 0.15	0.004

### Diagnostic Performance of the Various Models

**Figure 3:**
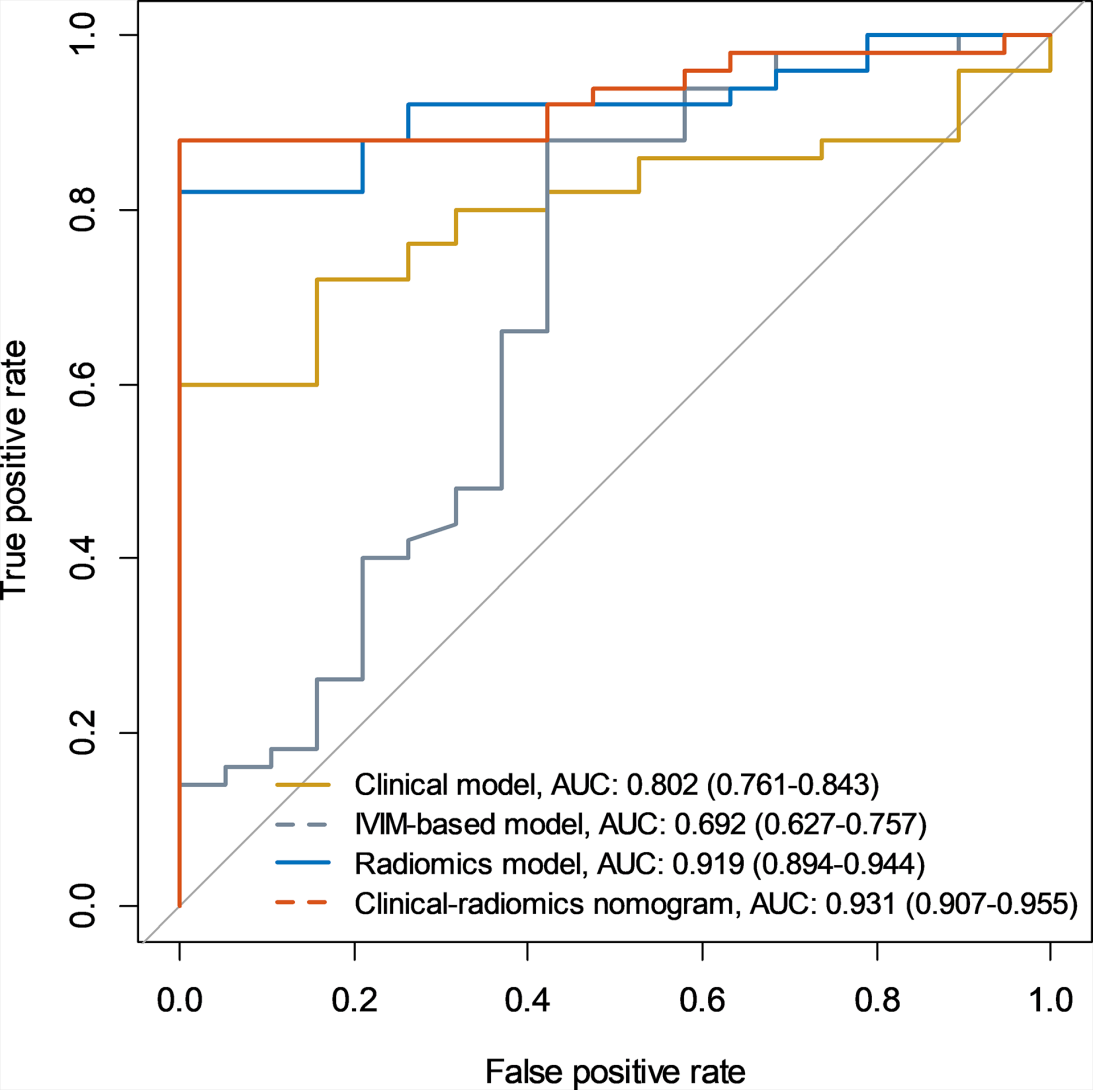
Receiver operating characteristic curves of the clinical model, IVIM-based model, radiomics model, and clinical-radiomics model.

Among the clinical vriables, only urine creatinine was an independent predictor of mf-AML (P=0.004). The clinical model based on urine creatinine achieved an AUC of 0.802 (95%CI: 0.761-0.843), with a sensitivity of 60%, specificity of 100%, and accuracy of 71% (**Table 3**). The IVIM-based model achieved an AUC of 0.692 (95%CI: 0.627-0.757), with a sensitivity of 66%, specificity of 63.2%, and accuracy of 65.2% (**Table 3** and **Figure 3**).

**Table 3:** The performance of various diagnostic models.

Models	AUC (95%CI)	Accuracy (%)	Sensitivity (%)	Specificity (%)
Clinical model	0.802 (0.761-0.843)	71.0	60.0	100
IVIM-based model	0.692 (0.627-0.757)	65.2	66.0	63.2
T2WI-radiomics model	0.883 (0.852-0.914)	84.1	82.0	89.5
IVIM-radiomics model	0.874 (0.841-0.907)	78.3	74.0	89.5
T2WI-IVIM-radiomics model	0.919 (0.894-0.944)	87.0	82.0	100
Clinical-radiomics model	0.931 (0.907-0.955)	89.9	88.0	94.7

**Figure 4:**
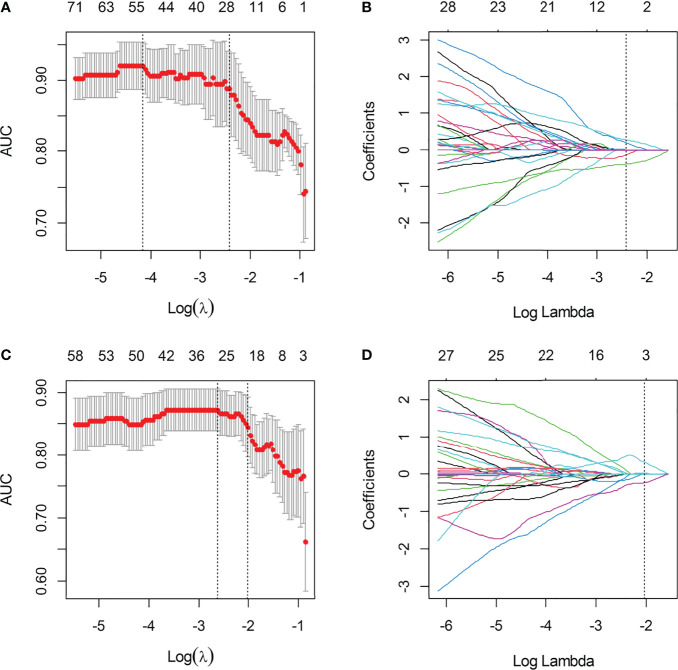
The selection of LASSO parameter for T2WI-radiomics and IVIM-radiomics models. **(A)** Select the optimal Log (λ) = -2.427 for IVIM; **(B)** Coefficient map of IVIM-derived radiomics features; **(C)** Select the optimal Log(λ) = -2.022 for T2WI; **(D)** Coefficient map of T2WI-derived radiomics features.

**Figure 5:**
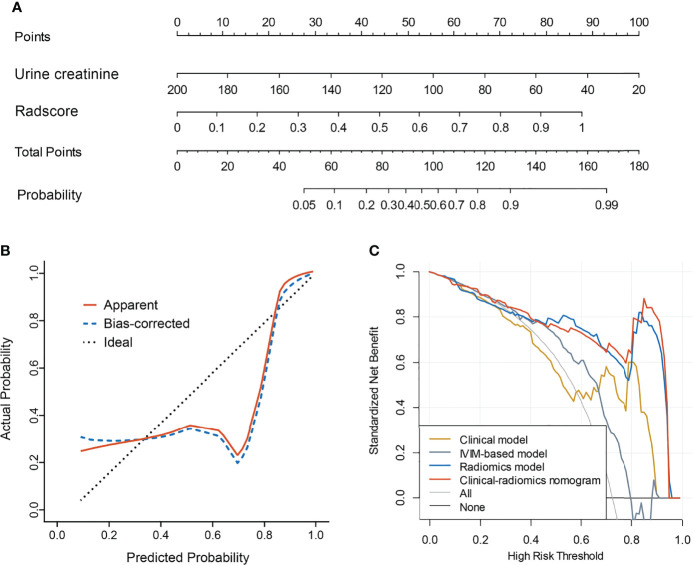
Clinical-radiomics model and its performance. **(A)** Nomogram based on urine creatinine and radscore; **(B)** Calibration curves for nomogram; **(C)** Decision curve analysis for clinical model, IVIM-based model, radiomics model, and the clinical-radiomics model.

A total of 396 extracted radiomic features from each sequence, 76.3% were stable. Finally, four and five features were selected for T2WI-radiomics and IVIM-radiomics models, respectively (**Figure 4** and **Table 4**). T2WI-radiomics and IVIM-radiomics model had an AUC of 0.883 (95%CI: 0.852-0.914) and 0.874 (95%CI: 0.841-0.907), respectively (**Table 3**). Radiomics model yielded a better performance (P<0.001), with an AUC of 0.919 (95%CI: 0.894-0.944), sensitivity of 82%, specificity of 100%, and accuracy of 87% (**Table 3** and **Figure 3**). Stepwise logistic regression of urine creatinine, *f* value, and radiomics score showed that only urine creatinine and radiomics score were retained as predictors. The clinical-radiomics model yielded an AUC of 0.931 (95%CI: 0.907-0.955), sensitivity of 88%, specificity of 95%, and accuracy of 90% (**Table 3** and **Figure 3**). The mean AUC of clinical-radiomics model after 1000 five-fold corss-validations was 0.927 (95%CI: 0.891-0.964). **Figure 5** **A** displays the clinical-radiomics nomogram for the evaluation of mf-AML probability. The Hosmer-Lemeshow test showed goodness of fit of the clinical-radiomics nomogram (P=383). The prediction rule showed good calibration between the observed and predicted probabilities in the clinical-radiomics nomogram (**Figure 5** **B**). In addition, the DCA graphically indicated that the clinical-radiomics model provided a large net benefit than other models over the relevant threshold range (**Figure 5** **C**).

**Table 4:** Selected radiomics features for T2WI-radiomics and IVIM-radiomics models.

Features	Coefficient
**T2WI-radiomics model**	
VoxelValueSum	0.202
ClusterShade_angle135_offset4	-0.131
Correlation_angle45_offset1	0.274
Compactness2	-0.383
SurfaceVolumeRatio	0.001
**IVIM-radiomics models**	
GLCMEnergy_AllDirection_offset1	0.028
InverseDifferenceMoment_angle90_offset4	0.317
ShortRunHighGreyLevelEmphasis_AllDirection_offset7_SD	-0.001
Maximum3DDiameter	-0.222

## Discussion

To the best of our knowledge, this present study is the first to distinguish mf-AML from RCC using a non-invasive MRI-based radiomics approach. We extracted radiomics features from conventional and functional MRI and compared their diagnostic performance. The results showed that the accuracy of the T2WI-radiomics was comparable to the IVIM-radiomics and their combination could achieve significant improvement. The addition of urine creatinine rather than IVIM parameters to the combined radiomics model further improved the diagnostic performance.

The mf-AML is often misdiagnosed as RCC prior to surgery (7). Differentiating these two entities is especially crucial for treatment planning and prognosis evaluation but challenging (28). At present, histopathology is the gold standard in the differential diagnosis of mf-AML and RCC. The mf-AML shares overlapping imaging features with RCC, making the differential diagnosis rather difficult by conventional imaging modalities (29). Among morphologic features, low signal intensity on T2WI favors mf-AML over RCC (8). Given the limited information recognized by conventional MRI, most of the previous studies used various DWI techniques to distinguish renal neoplasms. Tordjman et al. conducted a meta-analysis and the results showed that ADC of renal tumors excluding cystic and necrotic areas, provides better discriminatory ability than whole-lesion ADC to differentiate RCC from other renal lesions (11). Li et al. demonstrated that whole-tumor quantitative ADC histogram might be helpful for differentiation of mf-AML of RCC (12). The mf-AML had significantly lower ADC values than RCC (P<0.001). Li et al. found that water molecular diffusion heterogeneity index (α) from a stretched exponential model and true ADC (D) from a biexponential model resulted in improved differentiation with higher sensitivity and specificity between mf-AML and RCC compared with monoexponential ADC (15). In this present study, we explored the incremental value of IVIM to the T2WI in the discrimination between mf-AML and RCC. The results demonstrated that the combination of radiomics features derived from IVIM and T2WI enabled improvement in the diagnostic performance.

In recent years, radiomics is a specific field of medical research that has been used for the diagnosis, treatment response, and survival prediction of renal tumors (30–32). Radiomics analysis offers objective image information that could otherwise not be captured by radiologists’ subjective radiological interpretation. Overall, a recent review reported the superiority of radiomics over expert radiological assessment (33). Radiomics applications may support improved characterization of renal tumors. Radiomics is an attractive approach that has the potential to improve the non-invasive diagnostic accuracy of renal tumor imaging and the prediction of its natural behaviour. A majority of studies focused on the characterization of solid renal neoplasms (benign vs malignant) using MRI-based quantitative radiomics analyses (e.g., histogram and texture features) (34–36). However, only a small portion of studies aimed to distinguish subtypes of benign and malignant renal tumors, for instance, mf-AML and ccRCC (37–41). These studies proposed a CT-based radiomics model for preoperative differentiating mf-AML from homogeneous ccRCC (37–41). Ma et al. observed that mini-peritumoral and perirenal radiomics features contributed to the differentiation of mf-AML from ccRCC (38). We chose MRI instead of CT because the former provides multi-parametric sequences, which theoretically provides information than simple attenuation differences measured in Hounsfield units on CT (42). For the first time, we extracted radiomics features from T2WI and IVIM images and found that the diagnostic performance of both was similar, their combination could produce better discrimination of mf-AML and RCC. In contrast with radiomics, Xu et al. developed and validated an MRI-based deep learning model to differentiate benign from malignant renal tumors in clinic, and the model based on the combination of T2WI and DWI yielded the optimal performance (43). Xi et al. designed a deep learning model to distinguish benign from malignant renal lesions based on routine MR imaging with comparable performance as compared to experts and radiomics (42). However, the “black box” nature of deep learning is difficult to be interpreted and accepted by clinicians.

## Conclusions

Our study also has some limitations. Firstly, the incidence of mf-AML is significantly lower than that of RCC, which may induce a sample unbalanced problem. To avoid overfitting, we performed cross-validation. Secondly, we extracted two-dimensional (2D) features that might provide less tumor information than three-dimensional features. However, some previous studies showed that the predictive performance of features extracted from the maximum level of the tumor was higher than that of those features extracted from the whole tumor (44, 45). 2D features may increase the robustness of features compared with 3D features. Whether the sign of single layer can fully reflect the characteristics of kidney needs further confirmed. Thirdly, this study was conducted in a single-center with a small sample size, prospective multi-center validation is needed in the future. Finally, we didn’t explore the diagnostic value of the radiomics features extracted from IVIM maps based on other lower b values. However, these preliminary results may preclude further comparison.

In conclusion, the results of this study preliminarily showed that noninvasive differential diagnosis of RCC and mf-AML using radiomics has relatively high clinical significance based on MRI Image, it is worth further exploration and improvement in large cohorts to obtain a more mature differential diagnosis system to improve the diagnostic coincidence rate.

## Data Availability Statement

The original contributions presented in the study are included in the article/**Supplementary Material**. Further inquiries can be directed to the corresponding author.

## Ethics Statement

The studies involving human participants were reviewed and approved by Hunan Cancer’s Hospital. The patients/participants provided their written informed consent to participate in this study.

## Author Contributions

LJ and YL: conception and design. YL: provision of study materials or patients. YL, YX, HL, and SJ: collection and assembly of data. LJ, YL, and MY: data analysis and interpretation. All authors: manuscript writing and final approval of the manuscript. All authors contributed to the article and approved the submitted version.

## Funding

This work was supported by the science and technology innovation Program of Hunan Province (grant number: 2018SK50910).

## Conflict of Interest

Author HL was employed by the company GE Healthcare.

The remaining authors declare that the research was conducted in the absence of any commercial or financial relationships that could be construed as a potential conflict of interest.

## Publisher’s Note

All claims expressed in this article are solely those of the authors and do not necessarily represent those of their affiliated organizations, or those of the publisher, the editors and the reviewers. Any product that may be evaluated in this article, or claim that may be made by its manufacturer, is not guaranteed or endorsed by the publisher.
